# Effect of perfusion index on oxygen reserve index accuracy in estimating arterial oxygen tension in anesthetized dogs: Data reanalysis

**DOI:** 10.1371/journal.pone.0319823

**Published:** 2025-03-31

**Authors:** Francesca Zanusso, Luca Bellini

**Affiliations:** Department of Animal Medicine, Production and Health, University of Padova, Legnaro, Italy; Khalifa University of Science and Technology, UNITED ARAB EMIRATES

## Abstract

Multi-wave CO-oximetry, utilizing the oxygen reserve index (ORi), estimates arterial partial pressure of oxygen (PaO_2_) in mild hyperoxemia, between 100 and 200 mmHg, and concurrently quantifies local perfusion at the measurement site using the perfusion index (PI). This study explores how variations in PI influence the accuracy of ORi in estimating PaO_2_ in anesthetized dogs. Data from 37 mechanically ventilated dogs were retrospectively reanalyzed using a different approach. ORi and PI values were collected using a CO-oximeter. The data were categorized into four groups based on PI quartiles. In each group, the relationship between ORi and PaO_2_ was assessed using linear regression analysis, and the area under the receiver operating characteristic curve (AUROC) investigated the diagnostic performance of ORi in detecting PaO_2_ >  150 mmHg. Strong relationships between ORi and PaO_2_ were observed in groups with PI values <  2 (r^2^ ≥  0.63). The AUROC of ORi for identifying PaO_2_ > 150 mmHg decreased with PI >  2 compared to lower values (0.76 vs >  0.88). In this study, PI values >  2 negatively impacted ORi’s ability to estimate PaO_2_, likely due to fluctuations in blood flow perfusing the measurement site. The results of this study suggests that consideration of the PI value is essential when titrating oxygen therapy using ORi in anesthetized dogs.

## Introduction

Continuous assessment of oxygenation in anesthetized veterinary patients is essential to detect potentially life-threatening conditions [1]. While arterial blood gas analysis is the gold standard for measuring arterial oxygen content, its invasiveness and delayed results make it less practical, especially in small to medium-sized animals [2]. Pulse oximetry offers a non-invasive method to estimate oxygenation by measuring the percentage of hemoglobin saturated with oxygen in peripheral arterial blood (SpO_2_). This method is based on the relationship between arterial partial pressure of oxygen (PaO_2_) and oxygen-saturated hemoglobin (SaO_2_). Conventional pulse oximetry utilizes light wavelengths of 660 nm (red) and 940 nm (infra-red) to differentiate between oxyhemoglobin and deoxygenated hemoglobin [3]. However, the accuracy of pulse oximetry is influenced by the quality of arterial blood flow at the sensor site [4, 5], as measured by the perfusion index (PI) [6]. PI reflects the strength of peripheral pulse and varies with factors such as stroke volume and vascular tone [7]. Masimo’s Rainbow SET technology enhances pulse oximetry by incorporating CO-oximetry, which provides continuous feedback on perfusion status and signal quality. Studies in healthy humans have reported mean PI values ranging from 2.2 to 3.5 [8,9], though values can vary widely. In dogs under general anesthesia, PI values ranged from 0.3 to 1.9, influenced by factors such as tongue width and ambient light [10,11]. Ventilation mode and passive leg raising test also affects PI measurements [12,13]. Despite challenges like poor perfusion, modern pulse oximeters generally provide accurate SaO_2_ estimates even under conditions of hypoperfusion, hypotension, hypothermia, or vasopressors use [14]. In veterinary settings, probe placement can influence PI values and SpO_2_ readings [11].

Masimo Rainbow SET technology broadens measurement capabilities by assessing various hemoglobin forms and oxygen saturation in both arterial and venous blood, providing a more comprehensive view of oxygenation status. The oxygen reserve index (ORi), developed for human use, estimates the oxygen reserve by evaluating PaO_2_ levels from 100 to 200 mmHg [15–20]. Human studies have demonstrated a strong correlation between ORi and PaO_2_, though veterinary research shows varying correlations across species. A study on anesthetized donkeys found a mild correlation [21], whereas studies on dogs demonstrated a moderate correlation with ORi being effective in monitoring oxygen therapy [22,23,24].

A linear mixed-effects regression model showed that PI does not influence the correlation between PaO_2_ and ORi. However, the diagnostic performance of ORi in detecting PaO_2_ ≥  150 mmHg revealed high uncertainty at elevated PI values [22], suggesting a need for further research. The current study aims to reanalyze previously collected data to explore ORi’s effectiveness in estimating PaO_2_ changes across different PI ranges in anesthetized dogs and to identify the optimal PI value range for accurate ORi cut-off values for PaO_2_ ≥  150 mmHg. We hypothesized that local perfusion, as measured by PI, could have a significant impact on the correlation between ORi and PaO_2_, potentially influencing the accuracy and reliability of this relationship.

## Materials and methods

### Animals

The Ethics Committee of the University of Padova approved the study (OPBA 75/2021), and the dog owner gave consent before any procedures. In this retrospective analysis, simultaneous values of ORi, PaO_2_, and PI were analyzed from data collected by the authors in a previous study [22]. The study enrolled thirty-seven American Society of Anesthesiologists physical status I and II dogs anesthetized between October 2022 and May 2023, of both sexes (20 males and 17 females). Median (1^st^ quartile–3^rd^ quartile) age was 98 (73-122) months old and weight was 29.0 (20.0-33.5) kg, with a median body condition score of 6 (5-6) out of 9. The animals were admitted to the Veterinary Teaching Hospital, University of Padova, and underwent soft tissue surgeries or diagnostic imaging procedures under general anesthesia.

### Anesthesia

Dogs scheduled for diagnostic procedures received 0.1–0.2 mg/kg butorphanol (Dolorex; AnimalHealth Intervet Italia Srl, Segrate, Italy) alone or with 2–4 μg/kg dexmedetomidine (Dexdomitor; OrionCorporation, Espoo, Finland) intramuscularly as premedication before intravenous catheter placement. Methadone 0.1–0.2 mg/kg (Semfortan, DechraVeterinary Products Srl, Torino, Italy) was used for preemptive analgesia in animals undergoing surgical procedures. General anesthesia was induced with propofol (PropoVet; Zoetis, Roma, Italy) intravenously to effect, until orotracheal intubation was possible, followed by maintenance with sevoflurane (Sevorane; AbbVie 148 S.r.l., Campoverde di Aprilia, Italy) or isoflurane (Isoflo; Zoetis, Roma, Italy) carried in a mixture of oxygen and air to obtain a fraction of inspired oxygen (FiO_2_) between 0.21 and 0.50. After anesthesia induction, a 22G arterial catheter (Delta Med; Viadana, Italy) was inserted for blood pressure measurement and sampling. Mechanical ventilation with a pressure-control mode was started immediately after induction, and the settings were adjusted to maintain the end-tidal carbon dioxide pressure (EtCO_2_) between 35 and 45 mmHg. Parameters monitored during anesthesia and recorded at the time of arterial blood sampling included direct systemic arterial blood pressure (systolic [SAP], mean [MAP], and diastolic [DAP] pressure), EtCO_2_, FiO_2_, and temperature (Temp). All data were displayed on a multi-parameter monitor (Datex S/5; GE Healthcare; Helsinki, Finland).

### Measurements

Pulse trace and values of SpO_2_, ORi and PI were measured using a multiwavelength pulse CO-oximeter (Rad-97, Masimo Corp., Irvine, CA, USA), with the adhesive probe (RD Rainbow Lite SET-1 Neo; Masimo Corp., Irvine, CA, USA) wrapped circumferentially around the folded tongue and connected to the CO-oximeter. Values of PaO_2_ were measured by a blood gas analyzer (EDAN i15, EDAN, Shenzhen, China) using a multi-parameter cartridge (Test Cartridge BG10, EDAN, Shenzhen, China) after collecting a 1 ml blood sample collected through the arterial catheter into a pre-heparinized syringe (Pulset; Westmed Inc., AZ, USA). Prior to sampling, 2 ml of blood were drawn and discarded to prevent sample contamination or dilution. After sampling, the artery was flushed with 1 ml of heparinized solution (10 IU/ml). Coupled values of ORi and PaO_2_ were divided into four groups according to the value of PI. Since a physiologic range for PI is unavailable in both human and veterinary studies, in this study we arbitrary stratified the observation based on the four quartile ranges for the PI values. This approach relies on the median and interquartile range as a method to describe how data are distributed within a sample representative of the population. Values associated with PI values ≤  the first interquartile were included in group P1. Those with PI>  the first interquartile and ≤  the median were categorized into group P2. Values with PI>  the median and ≤  the third interquartile were allocated to group P3, while those with PI values>  the third interquartile were placed in group P4.

### Statistical analysis

The normal distribution of continuous variables was investigated using a Shapiro-Wilk test. Normally distributed values were presented as mean ±  standard deviation; otherwise, they were reported as median and interquartile range (IQR: 1^st^ quartile–3^rd^ quartile). Values of PaO_2_, ORi, hematocrit (Hct), PR (pulse rate), SAP, MAP, DAP, and Temp were compared among groups using a one-way ANOVA or Kruskal-Wallis test, as appropriate. Linear regression analysis, reporting the coefficient of determination (r^2^), was performed to investigate the linear relationship between all values of ORi and PaO_2_ in the entire dataset or within each PI group. The goodness of fit for the linear regression model in each PI group was evaluated using the F-statistic, as calculated by the lm() function, to determine the explanatory power of the model within different ranges of PI [25]. Receiver operating characteristic (ROC) curve analysis and the Youden Index were used to determine the optimal cut-off ORi value that identified a PaO_2_ >  150 mmHg within each PI group. For each cut-off, the associated sensitivity, specificity, positive predictive value, negative predictive value, the area under the curve (AUROC), and the 95% confidence interval (CI) were calculated. All statistical analyses were performed using RStudio (RStudio, PBC, Boston, 196 MA, US) as the interface for R (The R Foundation for Statistical Computing, Vienna, Austria). Significance was set at p <  0.05.

## Results

**Fig 1:**
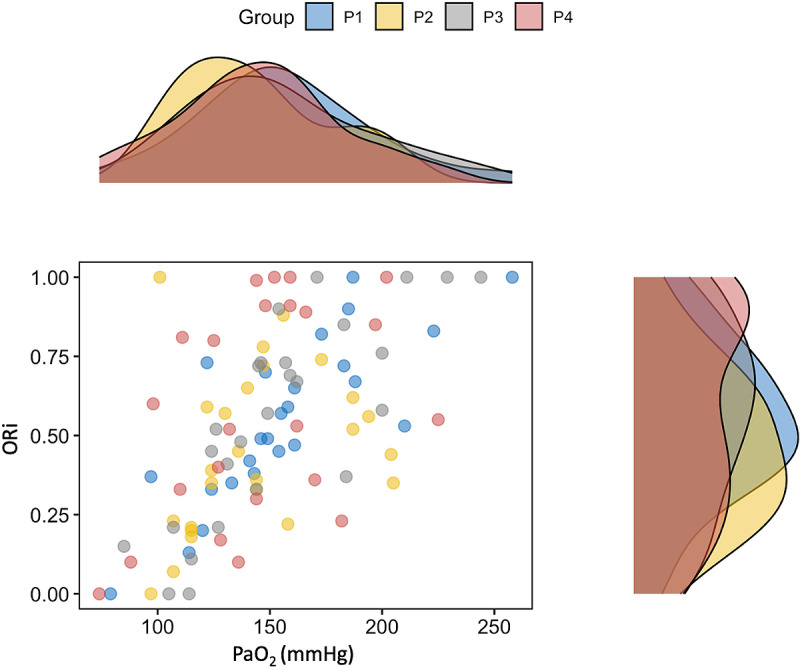
Dot plot and paired density plot of oxygen reserve index (ORi) and arterial partial pressure of oxygen (PaO_2_) in dogs. Groups P1, P2, P3, and P4 included measurements matching PI values between 0.23 and 0.79, 0.80 and 1.30, 1.40 and 1.90, and 2.00 and 3.40, respectively.

Among all paired values of ORi and PaO_2_ collected in the previous study [22], two were excluded because they did not have corresponding PI values. A total of 99 paired ORi, PaO_2_, PI, PR, SAP, MAP, DAP and Hct measurements were collected. Median ORi value was 0.53 (0.33-0.75), and mean PaO_2_ was 150 ±  37 mmHg. Median PI was 1.30 (0.80-1.90), and groups P1, P2, P3, and P4 included values of PI between 0.23 and 0.79, between 0.80 and 1.30, between 1.40 and 1.90, and between 2.00 and 3.40, respectively. Matched measurements of ORi and PaO_2_ were 25, 25, 26, and 23 in groups P1, P2, P3, and P4, respectively. Of the 37 dogs included in the study, 19 had ORi-PaO_2_ values that consistently corresponded to PI values within the same PI range. In contrast, for the remaining 18 dogs, the ORi-PaO_2_ values were paired with PI values that spanned different PI ranges. The distribution of ORi values varied slightly among the three groups (P1, P2, P3), displaying an almost bell-shaped pattern. In contrast, a flat distribution was observed in group P4 (Fig 1). No statistically significant difference was found in PaO_2_, ORi, Hct, PR, SAP, MAP, DAP, and Temp among groups (Table 1).

**Table 1:** Clinical data of dogs and comparisons among groups. Values are distributed into 4 groups based on the value of the perfusion index (PI). Groups P1, P2, P3, and P4 included measurements matching PI values between 0.23 and 0.79, 0.80 and 1.30, 1.40 and 1.90, and 2.00 and 3.40, respectively. Data are reported as mean ±  standard deviation or median and interquartile range (IQR). Statistical Significance was set for p <  0.05.

	P1	P2	P3	P4	p-value^*^
**ORi**	0.53 (0.38-0.72)	0.44 (0.23-0.62)	0.57 (0.34-0.75)	0.55 (0.32-0.90)	0.459
**PaO**_**2**_ (mmHg)	156.5 ± 39.5	144.2 ± 33.1	152.2 ± 38.6	145.2 ± 36.7	0.532
**Hct** (%)	35.0 (28.0-38.0)	36.0 (33.0-42.0)	38.0 (33.5-40.0)	34.0 (31.0-39.5)	0.319
**PR** (ppm)	109.0 (92.0-120.0)	85.0 (71.0-109.0)	82.5 (74.5-106.5)	90.0 (69.0-121.5)	0.076
**MAP** (mmHg)	84.0 (75.0-93.0)	79.0 (72.5-91.0)	75.0 (68.0-85.3)	75.0 (71.0-84.5)	0.380
**SAP** (mmHg)	114.3 ± 20.8	115.2 ± 22.4	111.2 ± 19.7	118.9 ± 33.2	0.694
**DAP** (mmHg)	62.0 (53.0-65.0)	64.0 (57.0-76.0)	57.5 (53.0-66.0)	63.0 (54.5-67.5)	0.361
**Temp** (C°)	36.0 (35.4-36.5)	36.6 (36.2-36.9)	36.5 (35.6-37.8)	37.0 (35.4-37.5)	0.122

ORi: oxygen reserve index, PaO_2_: arterial partial pressure of oxygen, PR: pulse rate, Hct: hematocrit, MAP: mean arterial blood pressure, SAP: systolic arterial blood pressure, DAP: diastolic arterial blood pressure, Temp: body temperature.

* calculated using a one-way ANOVA or Kruskal-Wallis test.

**Fig 2:**
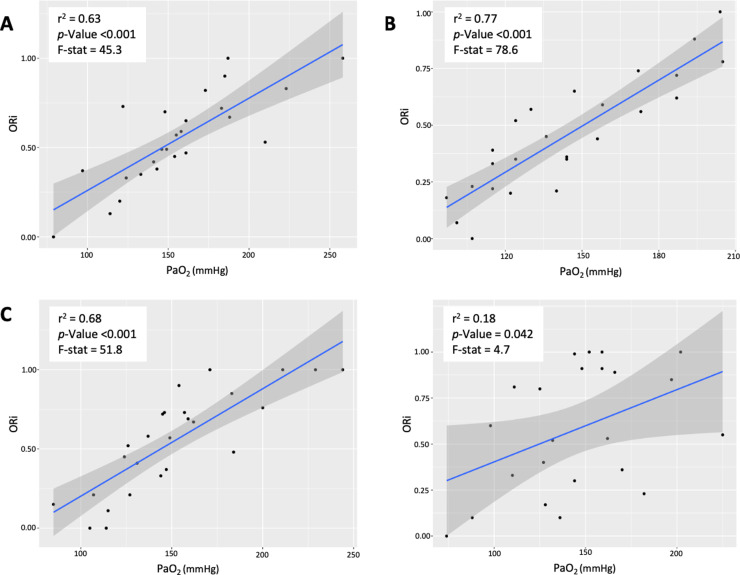
Coefficient of determination (r^2^) were assessed using linear regression analysis between paired values of oxygen reserve index (ORi) and arterial partial pressure of oxygen (PaO_2_) in groups P1 (A), P2 (B), P3 (C), and P4 (D). Groups P1, P2, P3, and P4 included measurements matching PI values between 0.23 and 0.79, 0.80 and 1.30, 1.40 and 1.90, and 2.00 and 3.40, respectively. F-stat: F-statistic.

A significant strong linear relationship between ORi and PaO_2_ values was found in groups P1, P2 and P3 (Fig 2). The relationship between ORi and PaO_2_ values in group P4 was very weak (r^2^ =  0.18, p =  0.04).

The Youden index identified ORi cut-off values of 0.53, 0.56, and 0.67, indicating PaO_2_ >  150 mmHg in groups P1, P2, and P3, respectively, with AUROC >  0.88, sensitivity >  0.85, and specificity >  0.83 (Table 2). In group P4, the ORi cut-off value indicating PaO_2_ >  150 mmHg was 0.85, with an AUROC (0.76) and a sensitivity (0.60) lower than that observed in the other groups.

**Table 2:** Diagnostic performance of oxygen reserve index (ORi) to detect arterial partial pressure of oxygen above 150 mmHg. Groups P1, P2, P3, and P4 included measurements matching PI values between 0.23 and 0.79, 0.80 and 1.30, 1.40 and 1.90, and 2.00 and 3.40, respectively.

Group	AUROC (CI 95%)	ORi cut-off	Sensitivity (CI 95%)	Specificity (CI 95%)	PPV (CI 95%)	NPV (CI 95%)
**P1**	0.88 (0.74-1.00)	0.53	0.85 (0.55-0.98)	0.83 (0.52-0.98)	0.85 (0.53-0.98)	0.83 (0.52-0.98)
**P2**	0.94 (0.86-1.00)	0.56	0.89 (0.52-1.00)	0.88 (0.62-0.98)	0.80 (0.48-0.99)	0.93 (0.65-0.99)
**P3**	0.94 (0.86-1.00)	0.67	0.91 (0.59-1.00)	0.87 (0.55-0.98)	0.83 (0.53-1.00)	0.93 (0.65-1.00)
**P4**	0.76 (0.56-0.96)	0.85	0.60 (0.26-0.88)	0.85 (0.55-0.98)	0.75 (0.40-0.94)	0.73 (0.40-0.96)

AUROC: area under the receiver operating characteristic curve, CI: confidential interval, PPV: positive predictive value, NPV: negative predictive value.

## Discussion

The main finding of this study indicates that measurements of ORi associated with a PI greater than 2 result in less accuracy in estimating PaO_2_, whereas higher accuracy is observed for PI range of 0.80-1.90. Within this range, the diagnostic capability of the oxygen reserve index in detecting arterial partial pressure of oxygen above 150 mmHg is maximized. The findings of the current study provide additional clinical insights and can complement the information presented by the authors in their initial assessment of ORi as a novel index that could enhance safety during anesthesia and in the early postoperative phase. Based on these results, the recommendation is to monitor PaO_2_ using an alternative device, such as a blood gas analyzer, if the CO-oximeter displays a PI outside of the specified range.

In dogs, ORi is an innovative and attractive parameter that has been investigated for non-invasively estimating oxygenation status in hyperoxemic patients [22,23]. Compared to standard pulse oximetry, which constantly registers SpO_2_ levels above 98% when PaO_2_ exceeds 100 mmHg, ORi was able to anticipate the decline in SpO_2_ during hyperoxemia in dogs [26]. Moreover, ORi demonstrated a good correlation with oxygen flow rate in sedated dogs [24], making it an appealing tool for monitoring and optimizing oxygen treatment. However, this also emphasizes the paramount importance of having a good accuracy for this device, as excessive hyperoxemia could lead to significant oxygen wastage and potentially result in atelectasis in dogs [27,28]. Moreover, in a study involving humans undergoing laparoscopic gastrectomy, the combination of ORi and SpO_2_ guided FiO_2_ adjustment reduced hyperoxemia compared to using SpO_2_ alone [29]. The clinical utility of ORi relies on its ability to estimate PaO_2_ within a reference range of 100 and 200 mmHg. Paired measurements of ORi and PaO_2_ exhibited a stronger correlation when the displayed PI was lower than 2. The increase in PI may reduce the accuracy of ORi in estimating PaO_2_, though the exact reasons remain unclear, based on the data collected in this study. PI reflects the ratio of pulsatile to non-pulsatile blood flow, so increases in pulsatile flow or decreases in non-pulsatile flow elevate PI. Vasodilation, often induced by inhalation anesthesia [30], raises PI levels, as seen in both humans and animals [31–35]. Additionally, external pressure from soft tissue or pulse oximeter probes can reduce non-pulsatile flow [36], further elevating PI and potentially affecting ORi reliability. In a study involving anesthetized dogs, inserting material between the tongue and the pulse oximeter probe resulted in significantly increased PI and a concurrent change in the recorded SpO_2_ [10]. In our study, using a pulse oximeter adhesive tape probe around the folded tongue may have led to tissue compression in some dogs. Furthermore, the probe position might have varied slightly among patients. In a study involving anesthetized dogs, PI values were higher at distances of 0.5 cm and 1 cm rostrally compared to the root of the tongue, significantly affecting the SpO_2_ output [11]. The difference in PI value could be attributed to the varying vascularization across different regions of the canine tongue, each with its distinct distribution of arteries, veins, and arteriovenous anastomoses. At the apex of the tongue, arteriovenous anastomoses are abundant, and external pressure applied to the tongue may compress them, potentially affecting the pulse oximeter signal. Mair and colleagues [11] observed higher SpO_2_ values with lower PI in anesthetized dogs using a Masimo Rainbow SET Pulse CO-oximeter. In contrast, in another study, the same authors [10] observed higher SpO_2_ values with higher PI using a Masimo SET Rad 5 pulse oximeter, but the lack of SaO_2_ measurements limits the interpretation of SpO_2_ accuracy based on PI in dogs. In humans, a recent study in healthy patients found a significant positive correlation between PI and SpO_2_, but not with ORi [37]. However, in this study, neither SaO_2_ nor PaO_2_ were measured to assess the accuracy of the device. These findings suggest only that there might be a relationship between the PI and signal acquisition by the pulse oximeter. It is important to note that the accuracy of SpO_2_ in estimating oxygen content may differ from ORi. While SpO_2_ readings primarily reflect arterial blood flow, ORi measurement is influenced by both arterial and venous blood. Consequently, PI, which accounts for both pulsatile and non-pulsatile blood flow, could exert a more significant impact.

In our study, PI also demonstrated an effect on the diagnostic ability of ORi in detecting mild hyperoxemia. Conversely, in a study involving critically ill patients, the diagnostic performance of ORi in detecting PaO_2_ levels above 100 mmHg appeared to be unaffected by the PI [38]. However, the authors reported a weak overall correlation (r =  0.13) between all ORi and PaO_2_ values, with a mean PI value of 2.9, notably higher than in our study. Additionally, the correlation between ORi and PaO_2_ across different PI values was not evaluated. In our investigation, we assessed the diagnostic capability of ORi in identifying PaO_2_ levels above 150 mmHg. We found that ORi’s diagnostic performance remains robust for PI values below 2, but it slightly diminishes for higher values, with a specificity of about 60%. This suggests that ORi is less effective in identifying individuals with hyperoxemia beyond 150 mmHg when the PI is above 2, while the sensitivity exceeds 83% across all PI values in our study. This suggests that ORi accurately identify patients with PaO_2_ levels below 150 mmHg, even when the PI exceeds 2. These findings underscore the utility of ORi in maintaining safe oxygen levels and promptly detecting critical deterioration in oxygenation status, as observed in human studies [39,40].

Our study showed some limitations. One was the method of stratifying paired data based on quartile ranges. Given the arbitrary nature of this choice, we cannot rule out the possibility that using a different grouping method, it could impact the outcomes, underscoring the need, in future studies, for a more accurate stratification approach. No information was available on the clinically relevant range of PI at which ORi estimates PaO_2_ with the highest accuracy. The authors decided to preliminarily investigate the diagnostic performance of ORi by dividing the sample according to the median and interquartile range. This is a descriptive data method used for reporting distribution of a variable in a population or sample. In future studies, narrower subgroups could more accurately determine the optimal interval of PI associated with the highest ORi accuracy in estimating PaO_2_. However, in the current study, increasing the number of PI ranges would result in subgroups with a limited number of observations, significantly decreasing statistical power.

Moreover, the sample size was not calculated, as the study is a reanalysis of previously collected data. However, in the retrospective sample size estimation, assuming an alpha of 0.05, a power of 0.9, and a coefficient of determination of 0.52 [22], the minimum number of paired measurements was estimated to be 12. Therefore, despite being a retrospective analysis, the number of values assigned to all PI groups exceeds the minimum sample size, ensuring sufficient statistical power.

In this study, several factors may have influenced the variability in PI readings. Inadequate probe contact or alignment and the use of a single-sized probe across dogs of varying body sizes could contribute to differences in PI, especially since tongue width was not measured. Over 50% of the dogs showed minimal PI variation, limiting their classification to a single PI group, which may have introduced subjective biases. The study focused on dogs with stable systemic hemodynamic conditions, but peripheral hypoperfusion at the probe site cannot be ruled out. In humans, PI values below 1.4 may indicate peripheral hypoperfusion [8], but no such cut-off exists for dogs, and the relationship between PI and poor peripheral perfusion in dogs is poorly understood. Additionally, the study only assessed local perfusion using PI values, neglecting other variables such as the core-to-toe temperature difference, which is correlated with perfusion [8]. The retrospective nature of the study introduces limitations, such as potential biases in data collection. A repeated measurement model was previously used to analyze the ORi-PaO_2_ correlation, but this approach did not account for individual animal effects. Despite these limitations, the study confirms that over 50% of the variability in ORi can be explained by PaO_2_, particularly when PI readings are below 1.9.

## Conclusions

This study highlights the pivotal role of PI values in influencing the accuracy of ORi readings for estimating PaO_2_ in anesthetized dogs. Elevated PI values (>2) can significantly decrease the ORi accuracy in estimating arterial oxygen levels. Clinicians should take into consideration the PI measurements displayed by the CO-oximeter when adjusting oxygen therapy based on ORi readings to optimize oxygen administration. Due to the retrospective nature of this study, further researches are warranted to confirm the ability of ORi to estimate PaO_2_ over a wide range of PI and to investigate how this may impact anesthesia outcomes.
